# Perioperative outcomes of robot-assisted versus video-assisted thoracoscopic surgery for non-small cell lung cancer: a meta-analysis focusing on real-world clinical studies in the past 10 years

**DOI:** 10.3389/fonc.2026.1835395

**Published:** 2026-05-18

**Authors:** Jianfang Zhu, Xiaopeng Meng, Liyong Hu

**Affiliations:** Department of Cardiothoracic Surgery, Shaoxing Second Hospital, Shaoxing, Zhejiang, China

**Keywords:** lymph node dissection, meta-analysis, non-small cell lung cancer, real-world study, robot-assisted thoracoscopic surgery, video-assisted thoracoscopic surgery

## Abstract

**Objective:**

Non-small cell lung cancer (NSCLC) accounts for more than 80% of lung cancer cases, and minimally invasive lobectomy is a standard curative approach for early-stage disease. The comparative perioperative outcomes of Robot-assisted thoracoscopic surgery (RATS) and Video-assisted thoracoscopic surgery (VATS) remain controversial. This meta-analysis compared perioperative outcomes of RATS and VATS for NSCLC using real-world clinical studies while excluding database-based analyses to better reflect routine practice.

**Methods:**

PubMed, Embase, the Cochrane Library, and Web of Science were searched for English-language studies published from January 2016 to December 2025. Eligible studies were real-world clinical reports directly comparing RATS with VATS in NSCLC; database analyses and clinical trials were excluded. Two reviewers independently screened studies and extracted data. Study quality was assessed using the Newcastle–Ottawa Scale (NOS). Meta-analysis was performed with Review Manager 5.3.

**Results:**

Nine studies were included (7 high-quality and 2 moderate-quality), involving 2, 130 patients with NSCLC (RATS: 1, 090; VATS: 1, 040). Pooled analyses showed no significant differences between RATS and VATS in intraoperative blood loss, postoperative length of stay, or postoperative complication rates. RATS was associated with a higher lymph node yield than VATS (weighted mean difference, +2.38). In the lobectomy subgroup, operative time and postoperative length of stay were shorter with RATS. Funnel plots did not suggest substantial publication bias.

**Conclusion:**

Based on real-world clinical evidence, RATS and VATS achieve comparable perioperative safety and short-term outcomes in NSCLC. RATS is associated with greater lymph node retrieval and may be a reasonable option when nodal yield is prioritized, particularly for patients undergoing lobectomy.

## Introduction

Non-small cell lung cancer (NSCLC) accounts for more than 80% of all lung cancer cases. Surgical resection remains the main curative treatment for early-stage disease, and minimally invasive approaches have become routine in thoracic surgical practice (1). Video-Assisted Thoracoscopic Surgery (VATS) has long served as the standard minimally invasive option for NSCLC, supported by its association with less operative trauma and faster postoperative recovery (2). Robot-assisted thoracoscopic surgery (RATS) has been used increasingly in recent years, aided by three-dimensional visualization and improved instrument dexterity, and has become an important alternative to VATS (3). However, differences in perioperative efficacy between the two approaches remain controversial (4). In real clinical settings, outcomes are also shaped by factors such as surgeon experience, institutional volume, and perioperative management pathways, but these sources of variability are not consistently accounted for in prior syntheses. Evidence that better reflects routine practice is therefore needed to support clinical decision-making.

Recent real-world studies comparing RATS and VATS in NSCLC have reported inconsistent findings. Some studies suggest that RATS is associated with lower intraoperative blood loss and more extensive lymph node dissection, while also incurring higher hospitalization costs and showing variable results regarding postoperative pain (5). Other studies have reported similar perioperative safety and short-term outcomes between the two techniques (6). A meta-analysis by Ma J et al. compared VATS and RATS (7); however, database-derived studies were included, which may limit the applicability of the pooled results to everyday clinical practice.

Against this background, we conducted a more restrictive meta-analysis that excluded database-related studies and focused on real-world clinical evidence. We limited inclusion to English-language studies to support consistent assessment and reduce heterogeneity related to reporting, and restricted the study period to the past 10 years (2016–2025) to reflect contemporary surgical practice. This study aims to clarify perioperative differences between RATS and VATS for NSCLC and to provide evidence that can assist individualized selection of minimally invasive surgical approaches in clinical practice.

## Materials and methods

### Study design

This study was conducted in accordance with the Preferred Reporting Items for Systematic Reviews and Meta-Analyses (PRISMA) guidelines and systematically compared perioperative outcomes between RATS and VATS for NSCLC from January 2016 to December 2025. To better reflect routine clinical practice, database-built summary analyses and data-mining studies were not included; only original real-world clinical studies were analyzed. In this review, “real-world clinical studies” referred to original patient-level clinical studies conducted in routine care settings, including single-center or multicenter retrospective or prospective observational cohorts and case-control studies with extractable perioperative data. Studies based primarily on administrative databases, national registries, or other secondary data platforms were excluded because they did not allow the same level of verification of case selection, perioperative management, and outcome definitions. A completed PRISMA 2020 checklist is provided in the **Supplementary Material**.

### Search databases

Four major English-language medical databases were searched: PubMed, Embase, Cochrane Library, and Web of Science. The search period was January 1, 2016, to December 31, 2025, and eligibility was restricted to English-language studies published within this timeframe.

### Search strategy

A combination of MeSH terms and free-text keywords was used. Core terms included “non-small cell lung cancer”, “NSCLC”, “robot-assisted thoracoscopic lobectomy”, “RATS”, “video-assisted thoracoscopic lobectomy”, “VATS”, “clinical study”, “real-world study”, “case series”, and “retrospective study”. The search syntax was adjusted to the rules and indexing features of each database to maximize retrieval of relevant studies. The full electronic search strategies for all databases, including the complete PubMed search string, are provided in the **Supplementary Material**.

### Inclusion criteria

Study type: English-language real-world clinical studies, including retrospective cohort studies, prospective cohort studies, and case-control studies. Studies were required to report complete clinical case data and were excluded if they involved database-based analytic datasets. Study subjects: Patients with NSCLC confirmed by pathological examination who underwent only simple RATS or VATS lobectomy without additional surgical procedures, with complete baseline information available (including age, gender, pathological type, tumor stage, etc.). Control group: Studies had to clearly define a RATS group (experimental group) and a VATS group (control group), and baseline characteristics between groups needed to be comparable without obvious selection bias. Outcome indicators: Studies were required to report at least one of the prespecified perioperative outcomes, including operation time, intraoperative blood loss, number of lymph nodes dissected, postoperative hospital stay, and postoperative complication rate (such as pulmonary infection, pneumothorax, hemorrhage, etc.). Publication time: January 1, 2016, to December 31, 2025; full text available with complete and extractable data.

### Exclusion criteria

Patients with other malignant tumors, severe heart, liver, kidney and other organ dysfunctions, coagulation disorders, and a history of thoracic surgery were excluded. Studies were excluded if outcome data were missing, not extractable, clearly incorrect, or logically inconsistent. Duplicate publications were excluded; when overlapping cohorts were reported, only the most recent and most complete report was retained.

### Screening process

Two trained reviewers independently completed literature screening. Titles and abstracts were first reviewed to exclude studies that clearly did not meet the inclusion criteria. Full texts of potentially eligible studies were then assessed in detail against the inclusion and exclusion criteria. Disagreements were resolved through discussion; if consensus could not be reached, a third senior reviewer adjudicated. The screening process was documented in detail, and a flow diagram was prepared to ensure transparency and traceability.

### Outcome indicators

Operation time: Defined as the total time from skin incision to completion of wound closure. Intraoperative blood loss: Defined as the total estimated blood loss during surgery, reported in milliliters. Number of lymph nodes dissected: Defined as the total number of lymph nodes retrieved during surgery and confirmed by pathological examination. Postoperative hospital stay: Defined as the time from the day of surgery to the day discharge criteria were met and discharge procedures were completed. Postoperative complications: Defined as surgery-related events occurring within 30 days after surgery, mainly including pulmonary infection, pneumothorax, pleural effusion, wound infection, hemorrhage, arrhythmia, etc.

### Data extraction

A standardized data extraction form was used. Two reviewers independently extracted study characteristics (first author, publication year, study country), baseline patient information (sample size, age, gender ratio, pathological type, tumor stage), surgical information (surgical approach, surgical timing), and perioperative outcomes. For continuous outcomes, means and standard deviations were extracted; for dichotomous outcomes, event counts and incidence were recorded. Information required for study design classification and quality assessment was also collected. Extracted data were cross-checked between reviewers. When key information was missing or unclear, corresponding authors were contacted when possible; unresolved issues were reviewed by a third reviewer to ensure accuracy and completeness.

### Quality evaluation

Quality assessment was performed using the Newcastle–Ottawa Scale (NOS) for observational studies. The NOS evaluates selection of participants, comparability between groups, and outcome assessment across eight items, with a maximum score of 9 points. Studies scoring 7–9 were considered high quality, 4–6 moderate quality, and 0–3 low quality. Two reviewers scored studies independently, and disagreements were resolved by a third senior reviewer.

### Statistical analysis

Review Manager 5.3 was used for data synthesis, with α=0.05. Heterogeneity across studies was assessed using the I² statistic. A fixed-effects model was used when heterogeneity was low (I²<50%). When heterogeneity was substantial (I²≥50%), clinical and methodological sources of variability were examined first, and subgroup analysis was undertaken only when a relevant variable was reported with sufficient consistency across studies; otherwise, a random-effects model was applied. Continuous outcomes were summarized as weighted mean differences (WMDs) with 95% confidence intervals (95% CIs), and dichotomous outcomes as odds ratios (ORs) with 95% CIs. Funnel plots were used to visually assess potential publication bias. Because fewer than 10 studies were available for each pooled outcome, formal statistical tests for funnel-plot asymmetry, such as Egger’s test, were not performed because their results would have been unstable and difficult to interpret.

## Results

### Literature screening results

**Figure 1:**
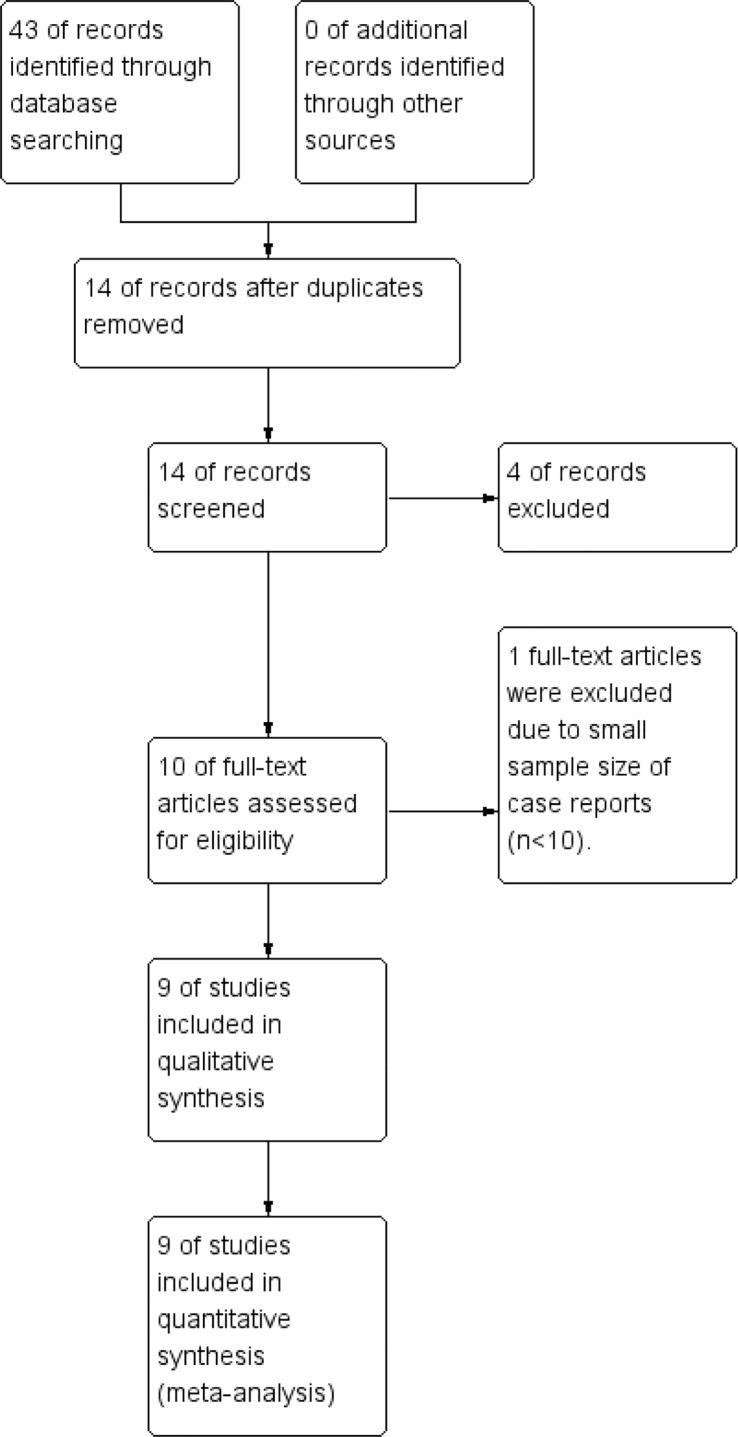
Flow diagram of literature screening and selection process.

The search identified 43 records. After screening titles and abstracts, studies that did not meet the inclusion criteria were excluded, including 8 non-English publications, 6 reviews/meta-analyses, 10 studies involving database analyses or data-mining approaches, and 5 clinical trials. Full texts of the remaining 14 studies were assessed. Three studies were excluded because key data were incomplete or not extractable, one study was excluded due to inadequate group comparability, and one small-sample case report (sample size <10) was excluded. Finally, 9 studies met all eligibility criteria and were included (8–16) (**Figure 1**).

### Baseline data of included studies

The 9 included studies comprised 2, 130 patients, including 1, 090 in the RATS group and 1, 040 in the VATS group. Two studies focused on segmentectomy, two included both lobectomy and segmentectomy, and the remaining studies primarily involved lobectomy (**Table 1**).

**Table 1:** Baseline characteristics of included studies and patients in RATS and VATS groups.

Author	RATS						VATS					
	n	Age	Male	Female	Lobectomy	Segmentectomy	n	Age	Male	Female	Lobectomy	Segmentectomy
Bao F 2016	69	58.6	26	43	62	7	69	59.9	22	47	62	7
Haruki T 2020	49	70	21	28	31	18	49	68	24	25	35	14
Kneuertz PJ 2020	245	65.3	47	53	245	–	118	64.6	45	55	118	–
Li C 2019	36	57.2	17	19	36	–	85	59.7	38	47	85	–
Li JT 2019	230	55.6	76	154	230	–	230	56	80	150	230	–
Merritt RE 2019	114	64.82	46	68	114	–	114	62.52	49	65	114	–
Qiu T 2020	40	61.4	36	4	40	–	38	61.7	34	4	38	–
Zhang Y 2020	257	53.53	84	173	–	257	257	52.21	89	168	–	257
Zhou Q 2020	50	54.7	15	35	–	50	80	57.7	26	54	–	80

### Literature quality evaluation

No low-quality studies were identified. Seven studies scored 7–9 on the Newcastle–Ottawa Scale (NOS) and were classified as high quality. These studies generally reported clear eligibility criteria, showed acceptable baseline comparability between groups, and provided complete perioperative outcome data (e.g., operative time and blood loss), without obvious evidence of major selection or measurement bias. Two studies scored 4–6 and were classified as moderate quality; the main limitation was that the basis for sample size estimation was not clearly described in the selection domain, while other domains were largely adequate. Overall, the included studies were of sufficient quality for quantitative synthesis (**Table 2**).

**Table 2:** Quality assessment of included studies using the Newcastle-Ottawa Scale (NOS).

Author	NOS
Bao F 2016	7
Haruki T 2020	7
Kneuertz PJ 2020	8
Li C 2019	5
Li JT 2019	7
Merritt RE 2019	7
Qiu T 2020	6
Zhang Y 2020	7
Zhou Q 2020	8

### Meta-analysis results

#### Comparison of operation time

**Figure 2:**
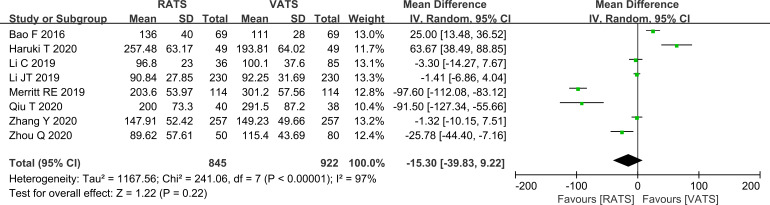
Forest plot comparing operative time between RATS and VATS groups (overall analysis).

Operative time was pooled first. Substantial heterogeneity was present (I2 = 97%), and a random-effects model was used. The pooled analysis showed no statistically significant difference in operative time between RATS and VATS (P>0.05, **Figure 2**). The substantial heterogeneity suggests that operative time was likely influenced by differences in case mix, institutional experience, and perioperative workflows across studies. Potential effects of study period and geographic setting should also be considered; however, given the limited number of studies and the uneven regional distribution of the included cohorts, further formal meta-regression or subgroup testing for these variables was not considered sufficiently robust.

#### Comparison of operation time in the lobectomy subgroup

**Figure 3:**
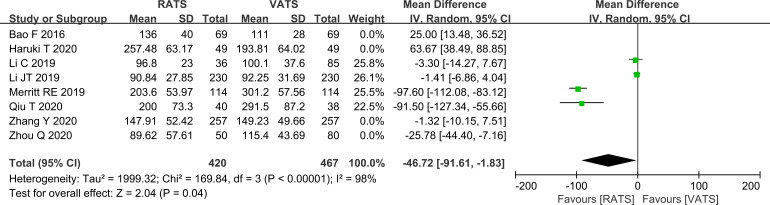
Forest plot comparing operative time between RATS and VATS groups (lobectomy subgroup analysis).

In the lobectomy subgroup, operative time was shorter in the RATS group than in the VATS group (P = 0.04, **Figure 3**).

#### Comparison of intraoperative blood loss

**Figure 4:**
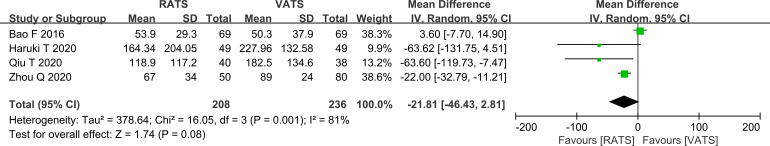
Forest plot comparing intraoperative blood loss between RATS and VATS groups.

Intraoperative blood loss was analyzed using a random-effects model. No statistically significant difference was observed between RATS and VATS (P>0.05, **Figure 4**). Only four studies reported blood loss, and a lobectomy-only subgroup would have included two studies; therefore, subgroup analysis was not performed.

#### Comparison of the number of lymph nodes dissected

**Figure 5:**
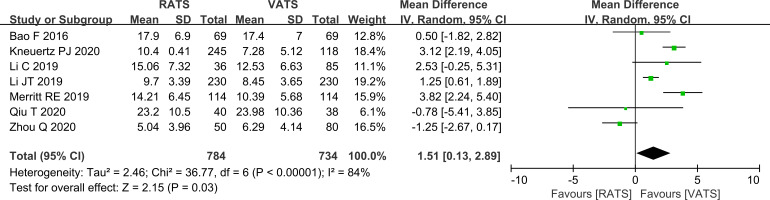
Forest plot comparing the number of lymph nodes dissected between RATS and VATS groups (overall analysis).

Lymph node yield was higher in the RATS group, with a pooled increase of 2.38 nodes compared with the VATS group (P<0.001, **Figure 5**).

#### Comparison of the number of lymph nodes dissected in the lobectomy subgroup

**Figure 6:**
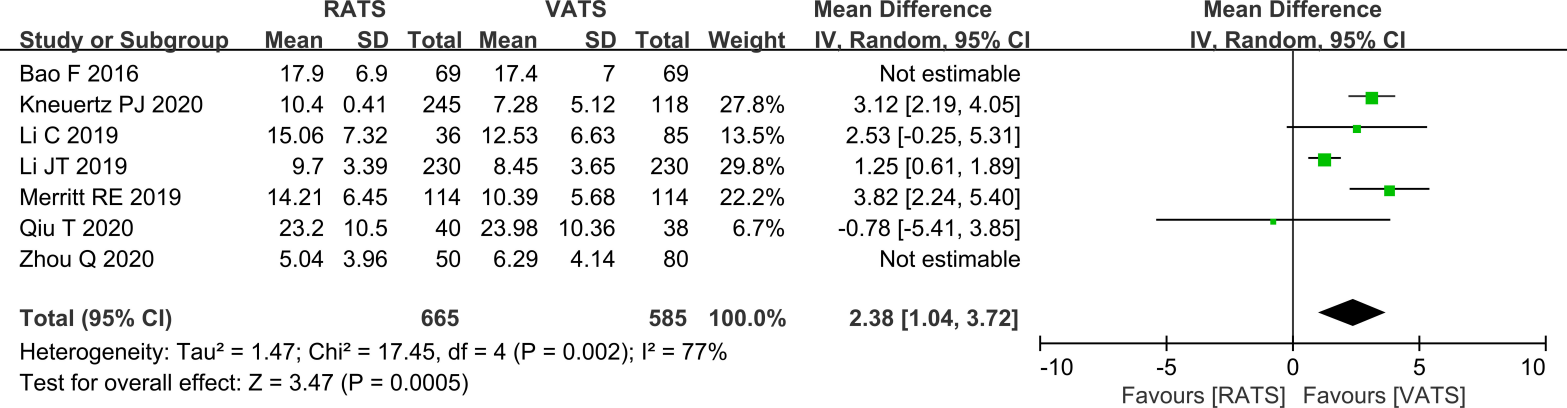
Forest plot comparing the number of lymph nodes dissected between RATS and VATS groups (lobectomy subgroup analysis).

The lobectomy subgroup analysis also showed higher lymph node yield in the RATS group (P<0.05, **Figure 6**).

#### Comparison of postoperative hospital stay

**Figure 7:**
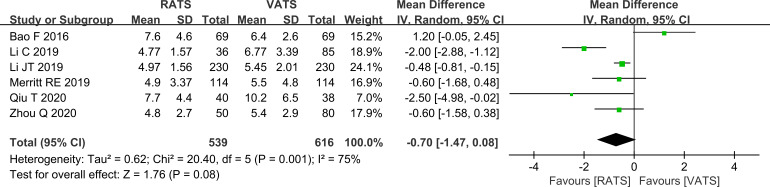
Forest plot comparing postoperative length of stay between RATS and VATS groups (overall analysis).

Pooled analysis of postoperative length of stay showed no statistically significant difference between the two groups (P>0.05, **Figure 7**) using a random-effects model.

#### Comparison of postoperative hospital stay in the lobectomy subgroup

**Figure 8:**
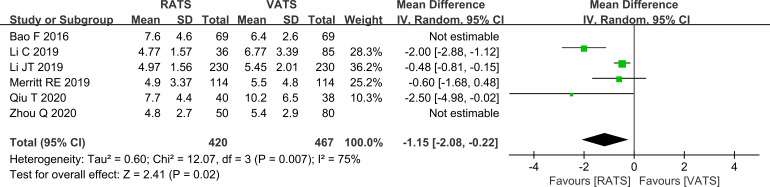
Forest plot comparing postoperative length of stay between RATS and VATS groups (lobectomy subgroup analysis).

In the lobectomy subgroup, postoperative hospital stay was shorter in the RATS group by 1.15 days compared with the VATS group (P = 0.02, **Figure 8**).

#### Comparison of safety

**Figure 9:**
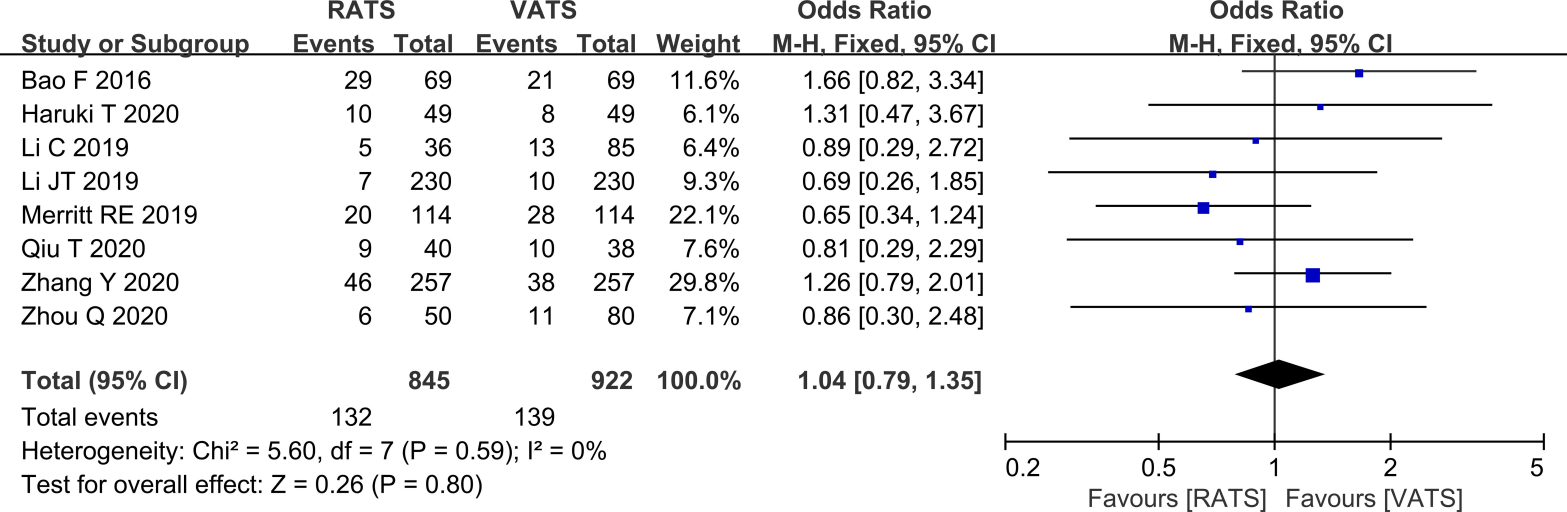
Forest plot comparing postoperative complication rates between RATS and VATS groups (overall analysis).

Postoperative complication rates did not differ significantly between RATS and VATS (P>0.05, **Figure 9**).

#### Comparison of safety in the lobectomy subgroup

**Figure 10:**
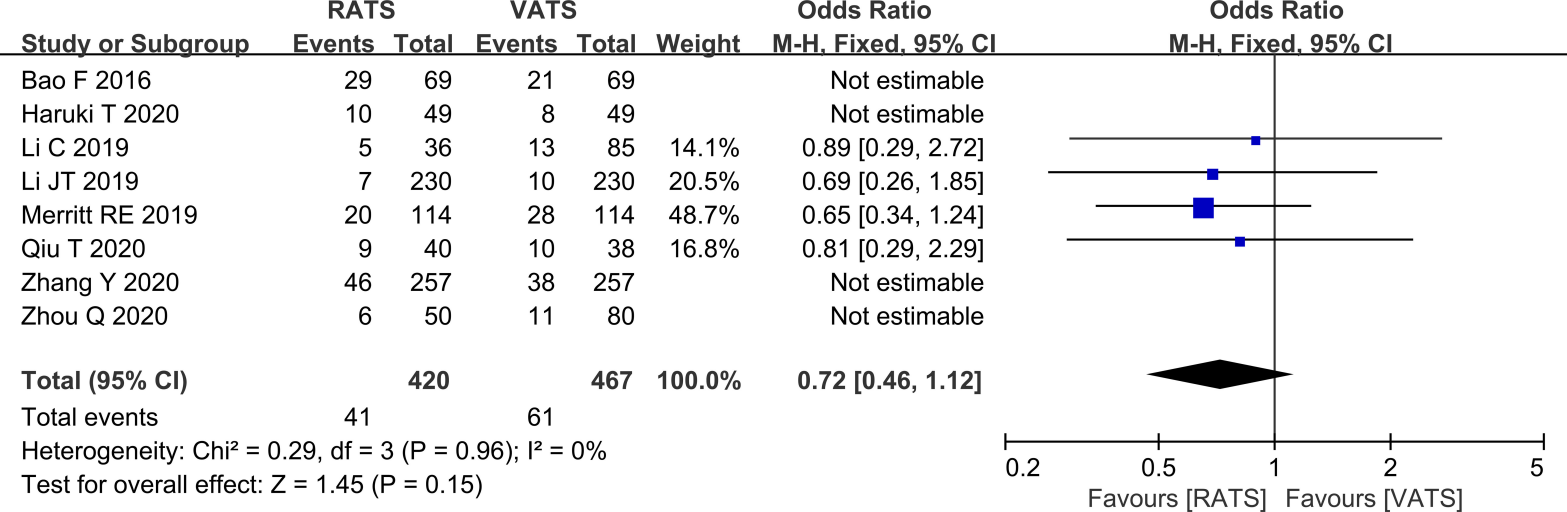
Forest plot comparing postoperative complication rates between RATS and VATS groups (lobectomy subgroup analysis).

In the lobectomy subgroup, postoperative complication rates remained similar between the two approaches (P>0.05, **Figure 10**).

### Publication bias analysis

**Figure 11:**
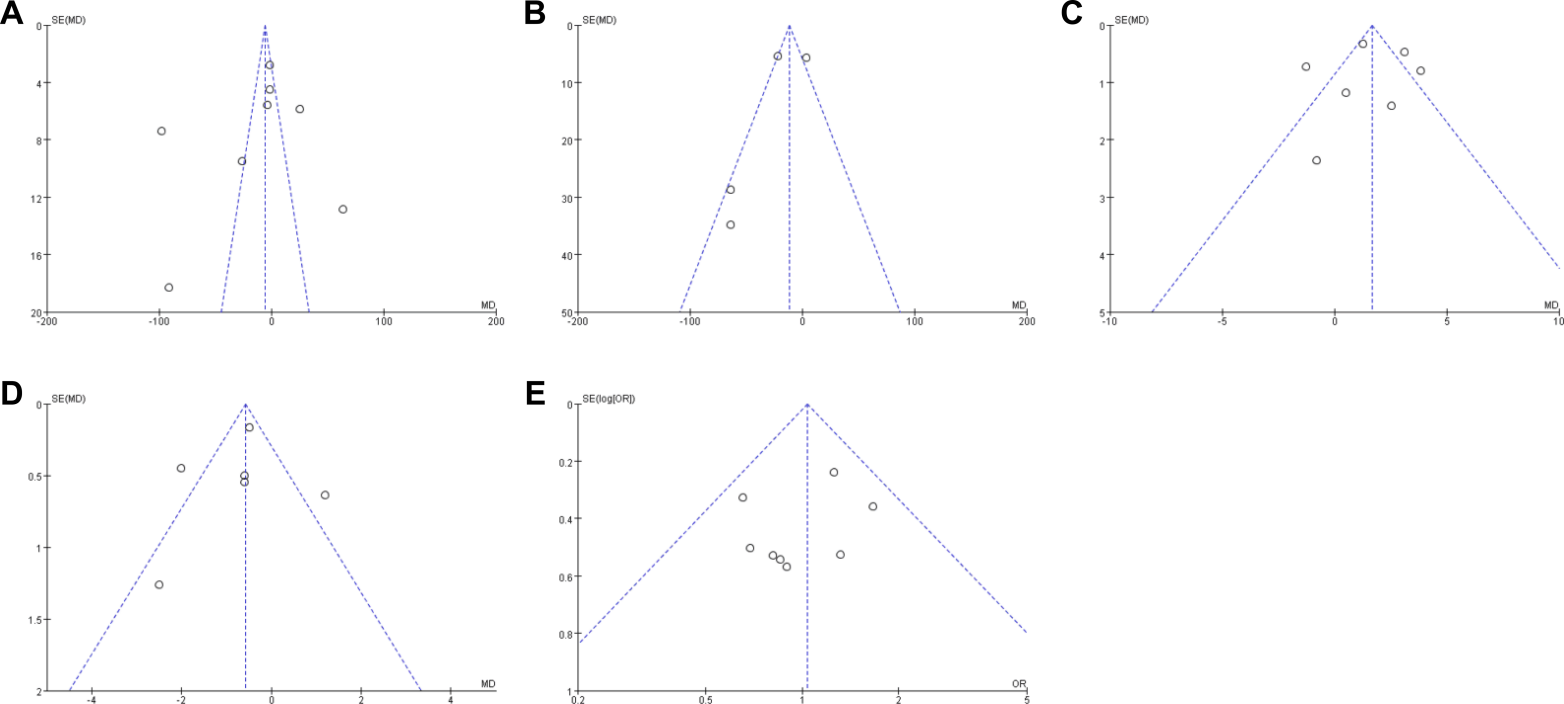
Funnel plots for assessing publication bias of main outcome indicators. **(A)** Operative time; **(B)** Intraoperative blood loss; **(C)** Number of lymph nodes dissected; **(D)** Postoperative length of stay; **(E)** Postoperative complication rate.

Funnel plots for the main outcomes appeared broadly symmetric, suggesting that substantial publication bias was unlikely (**Figure 11**).

## Discussion

This meta-analysis synthesized real-world clinical studies comparing perioperative outcomes of RATS and VATS in NSCLC. Overall, the two approaches showed similar perioperative safety and short-term recovery profiles, whereas RATS was associated with a higher lymph node yield. The main difference was lymph node yield, which was higher in the RATS group. In the lobectomy-only subgroup, RATS was associated with shorter operative time and shorter postoperative length of stay, while complication rates remained comparable.

Operative time did not differ in the overall analysis but differed in the lobectomy subgroup, a pattern that likely reflects the procedural mix across the included studies. Among the nine studies, two focused on segmentectomy, two included mixed cohorts of lobectomy and segmentectomy, and five evaluated lobectomy. Segmentectomy generally involves a more limited operative field, and VATS often provides sufficient visualization and maneuverability for key steps, which may reduce any efficiency advantage of RATS (17). Lobectomy, in contrast, requires careful handling of lobar vessels, bronchi, and regional lymph nodes (18). The three-dimensional view and greater instrument articulation available with RATS may facilitate precise dissection and ligation and reduce time spent on repeated instrument repositioning, which could explain the shorter operative time observed in the lobectomy subgroup (19). This interpretation is in line with trends reported in recent real-world cohorts (20, 21). The learning-curve effect should also be considered when interpreting these findings. Several real-world studies likely captured the early implementation phase of robotic thoracic surgery, when surgeons and operating-room teams were transitioning from established VATS workflows to RATS. During this phase, docking, console handling, and team coordination may prolong operative time independently of the intrinsic technical efficiency of the robotic platform. As institutional experience matures, this inflation in operative time may lessen.

Lymph node yield is a clinically relevant indicator of the extent of nodal assessment and the quality of perioperative staging in NSCLC (22). In this analysis, RATS was associated with a higher lymph node yield than VATS (pooled increase of 2.38 nodes), and this finding remained evident in the lobectomy subgroup. Because lymph node status directly affects pathological staging and subsequent management, incomplete nodal evaluation may increase the risk of understaging (23). From a technical perspective, the visualization and dexterity provided by RATS may improve access to anatomically constrained areas, including deeper mediastinal stations where exposure can be challenging with conventional thoracoscopy, potentially reducing missed nodal tissue (24). Given that the included studies reflect routine practice, this advantage in lymph node retrieval may be particularly relevant when thorough nodal assessment is emphasized. Although an additional 2.38 lymph nodes does not necessarily translate into upstaging in every cohort, more extensive nodal assessment may increase the detection of occult N1 or N2 disease. This stage-migration effect is clinically relevant because nodal upstaging can alter postoperative risk stratification and may increase the likelihood that adjuvant systemic therapy is recommended. Even though survival and adjuvant treatment data were not pooled in the present study, the higher nodal yield observed with RATS may still be meaningful by improving pathological staging accuracy and reducing the risk of undertreatment. For this reason, nodal retrieval may represent one of the most clinically relevant differences between the two approaches.

The lack of significant differences in blood loss, postoperative length of stay, and complication rates suggests that both approaches have comparable perioperative safety profiles in contemporary practice. VATS is well established and supported by standardized perioperative pathways in many centers, which may contribute to the convergence of short-term outcomes. Interpretation of blood loss should be cautious because only four studies reported this endpoint, limiting precision and preventing informative subgroup analyses. Postoperative complications were largely typical thoracic surgery events, such as pulmonary infection and pneumothorax, and their incidence did not differ between groups, indicating that RATS does not appear to increase short-term postoperative morbidity in real clinical settings (25).

Cost is also an important consideration in the real-world adoption of RATS. Although this meta-analysis did not pool economic outcomes, the present findings suggest a trade-off: RATS may offer a higher lymph node yield and, in lobectomy cases, a shorter postoperative hospital stay, whereas robotic acquisition, maintenance, and disposable costs are generally higher. In experienced, high-volume centers, some of the additional procedural cost may be partially offset by perioperative efficiency and shorter hospitalization, but this balance is likely to vary across healthcare systems and reimbursement models. Dedicated cost-effectiveness studies are therefore needed before broader economic conclusions can be drawn.

Heterogeneity was substantial for several endpoints, which is expected in observational real-world evidence. Differences in sample size, case mix, perioperative protocols, surgeon experience, and institutional adoption stage may all have contributed. For operative time in particular, variability in learning-curve effects, procedural composition, and team familiarity with robotic workflows is likely to be important. Subgroup analysis was therefore limited to surgical type, which was the most consistently reported clinical variable across studies; more granular stratification by tumor stage, surgeon experience, or institutional volume was not feasible because these data were not reported uniformly. Restricting analyses to lobectomy reduced some procedural heterogeneity, but residual variability remained. These findings should therefore be interpreted with appropriate caution. Differences in sample size, case mix, perioperative protocols, and surgeon experience across centers may all contribute. Variability in learning curves is particularly relevant for operative time, because robotic programs at different stages of adoption are unlikely to perform with the same efficiency. Differences in tumor stage and lesion location may also influence operative difficulty and recovery. Restricting analyses to lobectomy reduced procedural heterogeneity to some extent, but residual variability remained. Funnel plots for the main outcomes appeared broadly symmetric, suggesting that substantial publication bias was unlikely. Because fewer than 10 studies contributed to each pooled outcome, no formal statistical test for funnel-plot asymmetry was performed.

Several limitations should be acknowledged. Only nine studies met the eligibility criteria, which limits statistical power and may reduce the generalizability of the pooled estimates. In addition, our decision to exclude database-based studies and clinical trials was intended to improve clinical comparability and better reflect routine practice, but it may also have introduced selection bias by omitting other relevant evidence sources. More granular subgroup analyses by tumor stage, nodal status, surgeon experience, or institutional volume were not feasible because these variables were not reported consistently. Similarly, although study period and geographic region may have contributed to the heterogeneity in operative time, these factors could not be evaluated robustly because of the limited number of studies and the imbalance in regional representation. Restricting inclusion to English-language publications may have further contributed to regional bias. In addition, this review focused mainly on perioperative endpoints and did not synthesize several outcomes that are also relevant to clinical decision-making, such as conversion to thoracotomy, postoperative pain, chest tube duration, readmission, quality of life, long-term survival, recurrence, or detailed adjuvant treatment patterns. Accordingly, the present findings should be interpreted as evidence on short-term perioperative performance rather than as a complete basis for selecting one minimally invasive approach over the other.

## Conclusion

In real-world clinical studies, RATS and VATS show similar perioperative safety and short-term outcomes for NSCLC, with no clear differences in intraoperative blood loss, postoperative length of stay, or postoperative complication rates. RATS is associated with higher lymph node yield and, in lobectomy-focused analyses, may also shorten operative time and reduce postoperative hospitalization. Both approaches can be considered effective minimally invasive options, while RATS may be preferred when extensive nodal retrieval is a key priority. These findings are most informative for short-term perioperative decision-making and should be interpreted alongside long-term oncologic considerations, institutional expertise, and patient-specific priorities. Larger multi-center studies with standardized perioperative reporting are needed to confirm these findings and to better define which patient subgroups benefit most from each technique.

## Data Availability

Publicly available datasets were analyzed in this study. All data analyzed in this study were extracted from previously published, publicly available real-world clinical studies. No new datasets were generated. The source studies are cited in the reference list; all original data can be accessed through these published articles. No repository or accession numbers apply.
