# Supplementary material for: Perioperative outcomes of robot-assisted versus video-assisted thoracoscopic surgery for non-small cell lung cancer: a meta-analysis focusing on real-world clinical studies in the past 10 years
Source: Front Oncol. 2026 May 18;16:1835395. doi: 10.3389/fonc.2026.1835395 (PMC13222839; doi:10.3389/fonc.2026.1835395)
Supplement: Supplementary file 2 [file Supplementaryfile2.docx]

Supplementary File 1: Complete Search Strategies for All Databases

1. Embase Search Strategy (2011-01-01 to 2026-01-31, English only)

#1 'thoracolumbar fracture'/exp OR 'spinal fracture'/exp OR 'vertebral fracture'/exp

#2 (thoracolumbar fracture* OR spinal fracture* OR vertebral fracture*) AND (traumatic* OR unstable*)

#3 #1 OR #2

#4 'pedicle screw'/exp

#5 (percutaneous pedicle screw* OR PPSF OR minimally invasive pedicle screw* OR MIPSF)

#6 #4 OR #5

#7 'randomized controlled trial'/exp

#8 (randomized controlled trial* OR RCT* OR random* allocation* OR random* assignment*)

#9 #7 OR #8

#10 [lang]/en

#11 [pd]/2011-01-01 TO 2026-01-31

#12 #3 AND #6 AND #9 AND #10 AND #11

2. Cochrane Library Search Strategy (2011-01-01 to 2026-01-31, English only)

#1 MeSH descriptor: [Thoracolumbar Fractures] explode all trees

#2 MeSH descriptor: [Spinal Fractures] explode all trees

#3 MeSH descriptor: [Vertebral Fractures] explode all trees

#4 (thoracolumbar fracture* OR spinal fracture* OR vertebral fracture*) AND (traumatic* OR unstable*)

#5 (#1 OR #2 OR #3 OR #4)

#6 MeSH descriptor: [Pedicle Screws] explode all trees

#7 (percutaneous pedicle screw* OR PPSF OR minimally invasive pedicle screw* OR MIPSF)

#8 (#6 OR #7)

#9 MeSH descriptor: [Randomized Controlled Trial] explode all trees

#10 (randomized controlled trial* OR RCT* OR random* allocation* OR random* assignment*)

#11 (#9 OR #10)

#12 #5 AND #8 AND #11

#13 Filter: Language: English

#14 Filter: Publication date from 2011-01-01 to 2026-01-31

#15 #12 AND #13 AND #14

3. Web of Science Core Collection Search Strategy (2011-01-01 to 2026-01-31, English only)

#1 TS=(thoracolumbar fracture* OR spinal fracture* OR vertebral fracture*) AND TS=(traumatic* OR unstable*)

#2 TS=(percutaneous pedicle screw* OR PPSF OR minimally invasive pedicle screw* OR MIPSF)

#3 TS=(randomized controlled trial* OR RCT* OR random* allocation* OR random* assignment*)

#4 LA=English

#5 PY=2011-2026

#6 #1 AND #2 AND #3 AND #4 AND #5
